# Quality of life, psychological states, and personality traits in patients with pectus excavatum

**DOI:** 10.1016/j.xjon.2024.03.013

**Published:** 2024-04-02

**Authors:** Kohei Matsuda, Daisuke Fujisawa, Kyohei Masai, Naoki Miyazaki, Shigeki Suzuki, Yu Okubo, Kaoru Kaseda, Keisuke Asakura, Tomoyuki Hishida, Hisao Asamura

**Affiliations:** a Division of Thoracic Surgery, Department of Surgery, Keio University School of Medicine, Tokyo, Japan; b Department of General Thoracic Surgery, Sagamihara Kyodo Hospital, Kanagawa, Japan; c Department of Neuropsychiatry, Keio University School of Medicine, Tokyo, Japan; d Biostatistics Unit, Clinical and Translational Research Center, Keio University Hospital, Tokyo, Japan

**Keywords:** pectus excavatum, funnel chest, QOL, depression, social anxiety

## Abstract

**Objective:**

The quality of life (QOL) and psychological states of patients with pectus excavatum (PE) have yet to be well understood. This study aimed to evaluate the health-related QOL (HRQOL), psychological states, and personality traits of patients with PE, alongside the associations of these factors with the severity of PE.

**Methods:**

A cross-sectional evaluation was prospectively performed in patients scheduled to undergo PE repair surgery between July 2019 and April 2021. The primary outcome was the patients’ HRQOL, and the secondary outcomes were depression, social anxiety, self-efficacy, and personality traits.

**Results:**

In total, 129 patients were subjected to analyses. Patients' HRQOL had a lower role component summary score (mean ± standard deviation: 41.8 ± 12.8, *P* < .001) than the general population controls. Patients' HRQOL had a significantly better physical component summary (54.0 ± 10.4, *P* < .001) and mental component summary (53.3 ± 8.8, *P* < .001) than that of the general population. Fourteen patients' (10.9%) and 56 patients' (43.4%) scores indicated the presence of depression and social anxiety disorder, respectively. Patients’ self-efficacy (46.1 ± 11.4, *P*, .001) and level of extraversion (46.5 ± 11.8, *P* < .001) were lower than those of the general population. No significant correlation was found between the severity of PE and these scores.

**Conclusions:**

Our study revealed that patients with PE had decreased social-role QOL, depressive tendencies, increased social anxiety, lower self-efficacy, and introversion. No correlation between the severity of PE and the patients’ psychological outcomes leads us to conclude that surgical implications of PE should not be decided solely by a physical index.

## Graphical Abstract

**Figure:**
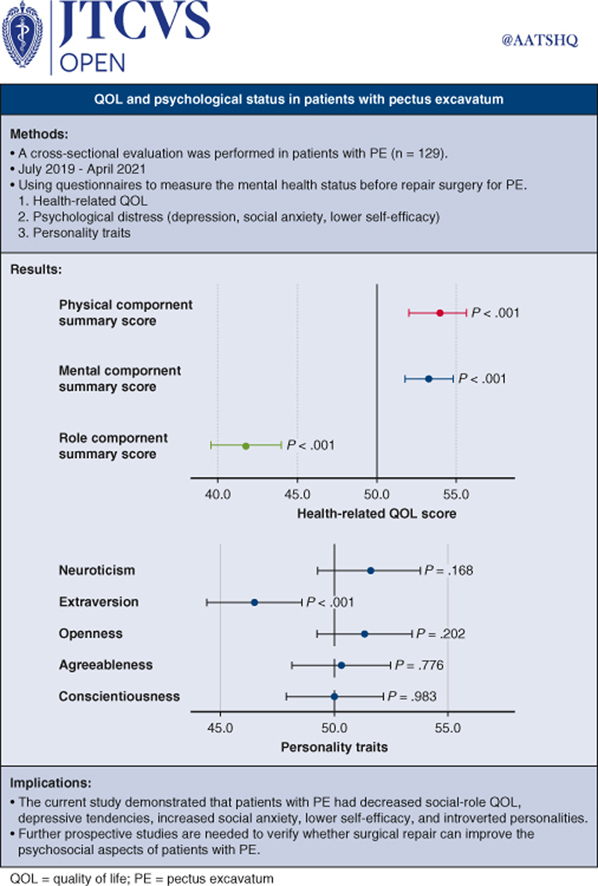

**Figure:**
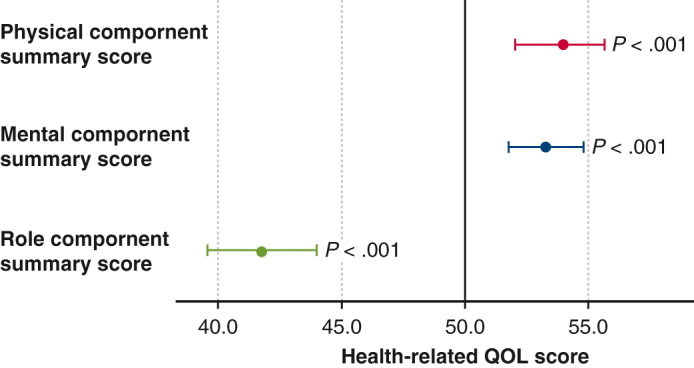
Cosmetic issues of PE lead to the patients' lowered social-role QOL.

**Central Message**

Regardless of the severity of PE, the patients had a decrease in social-role QOL. Cosmetic issues cause depressive tendencies, social phobia, lower self-efficacy, and introversion.

**Perspective**

The psychological aspects of patients with PE have not been investigated systematically. The current study demonstrated that patients with PE had decreased social-role QOL, depressive tendencies, increased social anxiety, lower self-efficacy, and introversion. Further prospective studies are needed to verify whether surgical repair can improve the psychosocial aspects of patients with PE.

Pectus excavatum (PE) is the most common congenital chest wall deformity, occurring in approximately 1 in every 400 to 1000 births. PE has various morphologies, with the deformity being deep, narrow, cup-shaped, broad, shallow, or deeply channel-shaped (Grand Canyon type).^1^ Patients with PE present with various physical symptoms, such as shortness of breath, palpitations, and chest pain, regardless of the degree of deformity.^2^^,^^3^

Imaging assessment using computed tomography (Haller index) is the most commonly used index to assess the severity of PE.^4^, ^5^, ^6^, ^7^ It is obtained by measuring the ratio of the widest transverse diameter of the chest (a) and the distance between the ventral spine and dorsal sternum (b) at the deepest point of the deformity (Haller index = a/b). Cardiac function evaluation using electrocardiography or echocardiography and lung function tests are also common methods to evaluate the severity of PE.^8^, ^9^, ^10^ Compression of the right heart attributable to severe depression of the chest wall and highly restrictive ventilatory impairment are good indicators for surgery.

Most patients with PE do not have significant physiological dysfunctions; rather, many patients seek surgical treatment for cosmetic reasons, which are often accompanied by lowered self-esteem, psychological distress, and affected social behaviors as a result. Psychological distress caused by the depressed morphology of PE is considered as the main indication for surgical treatment, although an objective evaluation of the psychological impact of PE is often difficult. Past studies report that approximately 80% of patients with PE are worried about their appearance and have a sense of self-denial, and 22.8% of patients have been bullied.^11^^,^^12^ Patients often experience prolonged social and mental distress due to the stigma by PE and anxiety as the result of precordial depression. Alleviation of these issues is also a crucial aspect of PE treatment alongside improving cardiopulmonary function. Since patients experience such distress from a very early stage of their life, the presence of PE is presumed to affect their personality traits, their ability to participate in vigorous physical activities, and their social and intimate relationships. However, to the best of our knowledge, psychological aspects of patients with PE have not been investigated systematically. Previous studies on this topic to date have been small in scale,^13^^,^^14^ with limited assessments, such as health-related quality of life (HRQOL).^15^ Therefore, in the current study, we aimed to comprehensively assess HRQOL, psychological status, and personality traits in patients with PE and their associations with the severity of PE.

## Methods

### Ethics Statements

This prospective cohort study was conducted following the principles of the Declaration of Helsinki and was approved by the institutional review board of Keio University School of Medicine, Tokyo, Japan (approval number: 20190027 on April 22, 2019). All patients provided written informed consent for the publication of their study data.

### Study Population

The eligible participants were all the patients who were scheduled to undergo repair surgery for PE deformities at Keio University Hospital, a university-affiliated tertiary medical facility located in central Tokyo, Japan. We excluded patients who did not agree with the contents of this study. The participants were consecutively recruited during the period between July 2019 and April 2021. A cross-sectional evaluation was performed, which will be described to follow.

### Assessments

The participants' clinical data, including age, sex, family history of PE, physical symptoms, radiologic findings (Haller index, asymmetrical depression, scoliosis), and cardiopulmonary function (electrocardiography and pulmonary function testing), were collected from their medical chart. The participants’ HRQOL (primary end point), their psychological distress (depression, social anxiety, and self-efficacy), and their personality traits were evaluated using the following questionnaires.

#### 12-Item Short Form Health Survey (SF-12)

The SF-12 is a 12-item self-reported questionnaire to measure the participants’ HRQOL. It has 8 subscales: (1) physical functioning, (2) role physical, (3) bodily pain, (4) general health, (5) vitality, (6) social functioning, (7) role emotional, and (8) mental health. From these subscales, 3-component summary scores (role component summary score [RCS], physical component summary score [PCS], and mental component summary score [MCS]) are calculated.^16^^,^^17^ The raw scores for all multi-item scales are transformed to norm-based scores of 0 to 100 and can be compared with the norm of the general Japanese population (mean values of 50 and a standard deviation of 10).

#### Patient Health Questionnaire 9 (PHQ-9)

The PHQ-9 is a 9-item self-reported instrument to measure the severity of patients’ depression.^18^ The total score of the PHQ-9 ranges from 0 to 27, with a greater score indicating greater severity of depression. The PHQ-9 scores are divided into 5 groups representing varying levels of depressive symptom severity: 0-4, minimal or none; 5-9, mild; 10-14, moderate; 15-19, moderately severe; and 20-27, severe. A total score of ≥10 indicates the presence of clinically significant depression.^19^

#### Liebowitz Social Anxiety Scale (LSAS)

The LSAS is a 24-item self-administered questionnaire to measure the participants’ level of social anxiety. The scale has 2 subscales: 13 items related to performance anxiety and 11 items related to social situations.^20^ The 24 items are first rated on a 0 to 3 scale regarding the fear felt in various social situations, and then those items are rated regarding the avoidance of the situation. Combining the total scores for the fear and avoidance sections provides an overall score with a maximum of 144 points. A total score of ≥44 indicates the presence of clinical social anxiety disorder in a Japanese sample (sensitivity, 93.3%; specificity, 90.0%).^21^

#### General Self-Efficacy Scale (GSES)

The GSES is a 16-item instrument to measure an individual's strength of general self-efficacy across a variety of everyday life settings by assessing optimistic self-beliefs to cope with various difficult demands in life.^22^ The scoring range is from 0 to 16. The raw scores are transformed to norm-based scores of 0 to 100 with mean values of 50 and a standard deviation of 10. The GSES scores are categorized into 5 groups representing varying levels of strength of general self-efficacy: ≤35, very low; 36-45, low; 46-55, average; 56-65, high; and ≥66, very high.

#### NEO Five-Factor Inventory (NEO-FFI)

The NEO-FFI is a 60-item self-report questionnaire to measure the participants’ personality traits. The questionnaire measures the “Big Five” dimensions of personality: neuroticism, extraversion, openness to experience, agreeableness, and conscientiousness. Each item is rated on a 5-point scale from “strongly disagree” to “strongly agree.”^23^ With the normative sample assuming a normal T distribution (mean, 50; standard deviation, 10), the results can be compared between the obtained and normative mean T values.

### Statistical Analysis

Data are expressed as frequencies and percentages for categorical variables and as mean and standard deviation for continuous variables. The participants' data were compared with the norm of the Japanese general population where applicable using the one-sample *t* test.^17^^,^^22^^,^^23^ The associations between the results of the questionnaires and the participants’ demographic and clinical characteristics (age, Haller index, vital capacity [VC], and forced expiratory volume in 1 second [FEV1.0]) were analyzed using the Pearson correlation analysis. Further, the results of the questionnaires were compared between the participants with and without symptoms, psychiatric disorders, electrocardiogram abnormalities, asymmetry depression, and scoliosis using *t* tests. Statistical analysis was conducted using the Statistical Package for the Social Sciences for Windows software (version 26.0; IBM Corp).

## Results

### Patients’ Characteristics

Of the 131 enrolled patients,2 patients did not consent to participation in the study and were excluded (Table E1). The remaining 129 patients were included in the analyses. The clinical characteristics of the included patients are summarized in Table 1. Most participants were male (108 patients, 83.7%), with a mean age of 24.6 years. Approximately one third of the participants had a family history of PE, and approximately one half of the participants had subjective symptoms, such as dyspnea on exertion and palpitations. Preoperative electrocardiography revealed significant findings in 63 (48.8%) patients. On pulmonary function testing, the average VC was 3850 mL and the average FEV1.0 was 3240 mL. Radiologic findings showed that the mean Haller index was 5.31. Sixty-three patients (48.8%) had asymmetrical PE with a mean sternal torsion angle of 19.7°. Ten patients (7.8%) had scoliosis, with a mean Cobb angle of 30.1°. Seven patients (5.4%) had psychiatric disorders.

**Table 1:** Patients’ clinical characteristics

Variables	Mean ± SD, n (%)
Number of patients	129
Age at surgery, y	24.6 ± 11.5
Sex	
Male	108 (83.7%)
Female	21 (16.3%)
Subjective symptoms	
Dyspnea on exertion	32 (20.9%)
Chest pain	15 (11.6%)
Chest tightness	11 (8.5%)
Palpitations	7 (5.4%)
Others	9 (7.0%)
Smoking	15 (11.6%)
Family history of pectus excavatum	39 (30.2%)
ECG abnormalities	63 (48.8%)
Right bundle branch block	29 (22.5%)
Right axis deviation	20 (15.5%)
Left atrium load	13 (10.1%)
First-degree atrioventricular block	2 (1.6%)
Pulmonary function testing	
VC, mL	3850 ± 930
FEV, mL	3240 ± 710
Haller index	5.31 ± 2.90
Asymmetry depression	63 (48.8%)
Sternal torsion angle, °	19.7 ± 11.2
Scoliosis	10 (7.8%)
Cobb angle °	30.1 ± 9.8
Psychiatric disorder	7 (5.4%)
Depression	5 (3.9%)
Schizophrenia	1 (0.8%)
Panic disorder	1 (0.8%)

*SD*, Standard deviation; *ECG*, electrocardiogram; *VC*, vital capacity; *FEV1.0*, forced expiratory volume in 1 second.

### Questionnaire Results

#### Health-related quality of life

**Figure 1:**
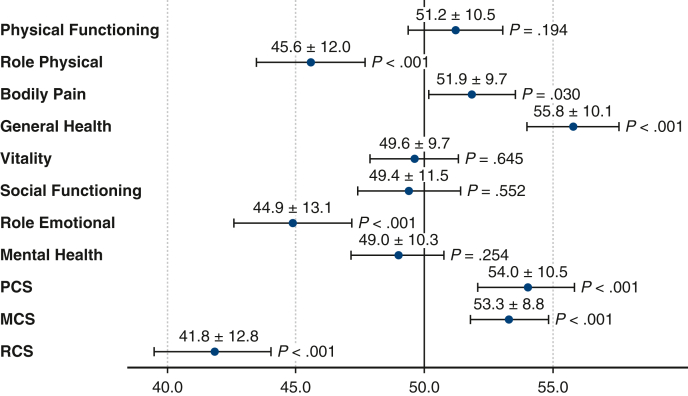
Descriptive analysis of the SF-12 subscales and summary scores. Patients had significantly lower scores for role physical, role emotional, and RCS than the norm-based Japanese population. They also had significantly greater scores for bodily pain, general health, PCS, and MCS than the norm-based Japanese population. *SF-12*, 12-Item Short Form Health Survey; *RCS*, role component summary score; *PCS*, physical component summary score; *MCS*, mental component summary score.

The participants' HRQOL, which was evaluated on the SF-12, is shown in Figure 1. The overall HRQOL of the patients with PE was significantly better than the norm-based Japanese population. The role component of the participants’ HRQOL was significantly lower (RCS: 41.8 ± 12.8, *P* < .001), whereas the physical and mental components were significantly greater (PCS: 54.0 ± 10.4, *P* < .001; MCS: 53.3 ± 8.8, *P* < .001) than the norm-based Japanese population. On each subscale, the patients with PE had significantly lower scores for role physical (45.6 ± 12.0, *P* < .001) and role emotional (44.9 ± 13.1, *P* < .001) domains and greater scores for bodily pain (51.9 ± 9.6, *P* = .029) and general health (55.8 ± 10.1, *P* < .001) than the norm-based Japanese population.

#### Depression

The participants' level of depression, which was measured on the PHQ-9, is summarized in Table 2. The participants’ mean level of depression was within normal range (mean PHQ-9 score: 4.51 ± 3.94). Fourteen patients (10.9%) scored ≥10 on the PHQ-9, which was indicative of depressive symptoms.

**Table 2:** Scores on mental state (PHQ-9, LSAS, and GSES)

	Mean ± SD, n (%)	*P* value	95% CI
PHQ-9	4.51 ± 3.94		
None (0-4)	75 (58.1%)		
Mild (5-9)	40 (31.0%)		
Moderate (10-14)	10 (7.8%)		
Moderately severe (15-19)	4 (3.1%)		
Severe (20+)	0 (0%)		
LSAS			
Total	42.6 ± 24.6		
Fear	23.6 ± 13.3		
Avoidance	19.0 ± 12.8		
GSES	46.1 ± 11.4	.001	44.1-48.0
Very low (≤35)	28 (21.7%)		
Low (36-45)	39 (30.2%)		
Average (46–55)	27 (20.9%)		
High (56-65)	27 (20.9%)		
Very high (≥65)	8 (6.2%)		

*SD*, Standard deviation; *CI*, confidence interval; *PHQ*, Patient Health Questionnaire; *LSAS*, Liebowitz Social Anxiety Scale; *GSES*, General Self-Efficacy Scale.

#### Social anxiety

The level of social anxiety, which was measured on the LSAS, is presented in Table 2. The patients’ mean total score was 42.6 ± 24.6; mean scores for the fear and avoidance subscales were 23.6 ± 13.3 and 19.0 ± 12.8, respectively. Fifty-six patients (43.4%) scored ≥44, which was indicative of social anxiety disorder.

#### Self-efficacy

The participants’ self-efficacy, measured on the GSES, is summarized in Table 2. The participants had significantly lower levels of self-efficacy (46.1 ± 11.4, *P* = .001) than the general Japanese population. Approximately 21.7% of the respondents were categorized as having a very low generalized self-efficacy level and 30.2% were categorized as having a low generalized self-efficacy level.

#### Personality traits

**Figure 2:**
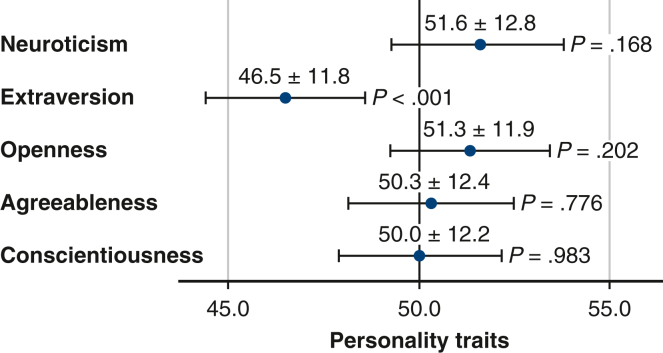
Descriptive analysis of the NEO-FFI scores. The extraversion score of the patient group was significantly lower than the mean score of the norm-based Japanese population. There were no significant differences between the groups in the other scale scores. *NEO-FFI*, NEO Five-Factor Inventory.

The participants' personality traits, as represented with the NEO-FFI, are summarized in Figure 2. The participants’ T score in the extraversion factor was significantly lower than the mean score of the norm-based Japanese population (46.5 ± 11.8, *P* < .001). No significant differences were observed between the patient and norm-based Japanese population in other personality factors.

### Correlations Between Questionnaire Results and Clinical Data

Although the correlation was relatively weak, Pearson correlation analyses demonstrated that the participants' age was negatively correlated with some aspects of the participants’ HRQOL (physical functioning, general health, vitality, mental health, and the mental component summary scores on the SF-12) and self-efficacy (GSES), and positively correlated with the neuroticism factor in the NEO-FFI (Table 3). No correlation was found between the Haller index and the questionnaire results (Table E2). Similarly, no significant differences were observed in the patients with and without symptoms (Table E3). In contrast, there were significant differences in the results of some questionnaires depending on whether a psychiatric disorder had been previously diagnosed (Table E4). The PHQ-9 scores were significantly greater for the patients with than those without psychiatric disorders. Three patients (42.9%) scored 10 or greater, which indicated clinical depression. The patients with psychiatric disorders scored worse for several domains of HRQOL (general health, mental health, and MCS), and self-efficacy. The level of extraversion, measured using the NEO-FFI, was lower in patients with psychiatric disorders than in those without.

**Table 3:** Correlation between age and questionnaire results

Parameters	r value	*P* value
SF-12		
Subscales		
Physical functioning	−0.178	.044
Role physical	0.015	.863
Bodily pain	−0.021	.810
General health	−0.308	<.001
Vitality	−0.206	.019
Social functioning	−0.040	.653
Role emotional	−0.096	.279
Mental health	−0.196	.026
Summary scores		
PCS	−0.125	.159
MCS	−0.275	.002
RCS	0.057	.524
PHQ-9	0.172	.051
LSAS	0.161	.068
GSES	−0.260	.003
NEO-FFI		
Neuroticism	0.218	.013
Extraversion	−0.114	.198
Openness	0.122	.17
Agreeableness	0.001	.992
Conscientiousness	−0.103	.243

*SF-12*, 12-Item Short Form Health Survey; *PCS*, physical component summary score; *MCS*, mental component summary score; *RCS*, role component summary score; *PHQ*, Patient Health Questionnaire; *LSAS*, Liebowitz Social Anxiety Scale; *GSES*, General Self-Efficacy Scale; *NEO-FFI*, NEO Five-Factor Inventory.

We investigated the correlation between the questionnaire results and physical findings, including respiratory function, electrocardiogram abnormalities, asymmetric depression, and scoliosis. Both VC and FEV1.0 had relatively weak positive correlations only with the openness factor in the NEO-FFI (Table E5). No significant differences were observed in the patients with and without electrocardiogram abnormalities (Table E6). The patients with asymmetry depression scored better in role physical on HRQOL and depressive symptoms (Table E7). Patients with scoliosis scored better in physical functioning on HRQOL and had greater conscientiousness according to the NEO-FFI (Table E8).

## Discussion

In the current study, the authors comprehensively evaluated the HRQOL, psychological states (depression, social anxiety, and self-efficacy), and personality traits of patients scheduled to undergo surgical treatment for PE. To the best of our knowledge, this is the first study to demonstrate a distinct social role impairment, high prevalence of social anxiety, and lowered extraversion tendency in this population.

A novel finding of the current study is that the patients with PE had distinctively low role-related QOL, whereas the physical and mental aspects of QOL were relatively high when compared with the general population. This contrasts with the findings of most previous studies, which have reported that patients with PE experience lower physical and psychological QOL.^24^, ^25^, ^26^ A plausible reason for this finding is that since the participants of the current study were young and had few comorbidities, they were considered to be generally healthy, except for PE, compared with the general population. In a previous report assessing HRQOL in patients with PE using the SF-36, Steinmann and colleagues^26^ reported that the PCS was lower in the patient group than in the control group, and there was no significant difference in the MCS between the groups. In Asian countries, the calculation method of the summary score is different from that of Western countries, and it is evaluated by a 3-component summary score, including the RCS.^27^ When evaluating patients using the 3-component summary score, the reduction in social QOL was more remarkable.

It is of note that 10.9% of the participants had a depressive tendency, defined by a total score ≥10 on the PHQ-9, and may need further psychological care. This prevalence was greater than that in a past Japanese study involving 1453 outpatients with cardiovascular diseases, which reported a prevalence of 5.6%.^28^ However, careful interpretation is needed, since the prevalence of depressive tendency measured with the PHQ-9 tends to vary widely. For example, in Japanese nonclinical samples with similar demographic backgrounds, the prevalence of depressive tendency ranged between 2.0% and 15.5%.^29^^,^^30^ The prevalence of clinical depression needs to be further investigated with more stringent criteria.

A distinctively high prevalence of social anxiety was observed in the current study. Approximately 43.4% of the participants scored ≥44 on the LSAS-J, which is indicative of the presence of social anxiety disorder. This finding is consistent with that of a previous study in which the adolescents aged 10 to 18 years with PE had significantly greater social anxiety scores than age-matched healthy controls.^25^

The participants of the current study reported significantly lower levels of self-efficacy than those in the norm-based Japanese population. This finding is consistent with those of past studies, which reported lowered level of self-esteem, self-assurance, and self-acceptance of their sample.^26^^,^^31^

The current study is one of the few studies that have examined the personality traits of patients with PE. We elucidated that the extraversion factor was significantly lower in the patient group than in the norm-based Japanese population, suggesting that patients with PE have distinct characteristics in a social context. Our results echo the findings of a study by Zhao and colleagues,^12^ which examined a total of 234 adolescent patients with PE and reported a heightened score of introversion on the Eysenck Personality Questionnaire.

In summary, patients with PE experience social-role dysfunction although their physical and general psychological states are intact. We speculate that cosmetic issues caused by PE led to the patients' lowered self-efficacy, heightened social anxiety, and heightened introversion, which resulted in reduced social participation (lowered social-role QOL). Considering that the patients' personality traits other than introversion were within the normal range, and that the level of depression in the sample was comparable with that of general population, we presume that the patients’ personality traits and social-role QOL could be recovered through PE surgery.

In the current study, no significant correlation was found between the severity of PE and the patients’ psychological outcomes. Findings on the correlation between the physical/functional and psychosocial aspects of PE have been inconsistent.^24^ However, the surgical repair of PE has been reported to lead to improved psychological aspects among patients with PE. Our findings imply that the surgical indications for PE should not be decided solely according to physical index; the psychological state and its effects on social functioning of the patients should also be considered.

Interestingly, there was a significant negative correlation between age and MCS, and a significant positive correlation between age and neuroticism. Two possible interpretations are considered. First, patients with PE experience stronger psychological distress and neurotic tendency as they grow older, and opportunities for social interaction increase with age. Alternatively, since the participants of this study were recruited from among patients who were willing to receive surgical repair of PE, patients with greater neurotic tendencies were more reluctant to receive a medical examination; therefore, they visited the surgical department at an older age. However, these correlations were relatively weak, and further verification is needed.

The patients with comorbid psychiatric disorders scored worse for several domains of HRQOL (general health, mental health, and mental component summary), depressive symptoms and self-efficacy than those without. They also had lower extraversion based on the NEO-FFI personality inventory. However, since a few patients had psychiatric disorders in this population, the impact of a prior psychiatric diagnosis was minimal. Significant associations were observed between a few psychometric variables and some of the physical findings, including respiratory function, asymmetric depression, and scoliosis. Further investigation on the mind–body association is warranted.

This study has a few limitations. First, this study is a single-center study, and the participants were limited to those who sought surgical treatment; thus, the generalizability of the study is limited. These results may not apply to other nations or cultures. The mean Haller Index of the current study (5.31 ± 2.9) was relatively greater than that reported by Steinmann and colleagues^26^ (4.4 ± 1.5), and there were several cases of asymmetric deformities. This may be attributed to our inclusion of only patients who sought surgical treatment. Second, the assessments of psychological states were based on self-administered questionnaires alone. Response bias may have been associated with social desirability and acquiescence, and some responses may have been incorrect because of the misunderstanding of the questions. However, we assured the patients of confidentiality and explained that the results of the questionnaire would not affect their treatment before the study. Clinical interviews by mental health professionals are necessary to confirm the clinical diagnosis of depression and/or social anxiety disorder. Third, this study did not include a control group. The results of our study were compared with those of standard values of the Japanese general population concerning the participants’ HRQOL, self-efficacy, and personality traits, and were compared with past studies concerning social anxiety and depression. The data were collected under different circumstances; our study period partly overlapped with the COVID-19 pandemic. Therefore, our results should be interpreted with caution. Fourth, the current study failed to evaluate some important psychosocial variables that may influence the HRQOL of patients with PE. For example, coping strategies, perceived social support, and body image concerns can affect the experiences of individuals with PE. Lastly, since this study had a cross-sectional design, causal relationships remain unknown, and further prospective studies are needed.

## Conclusions

**Figure 3:**
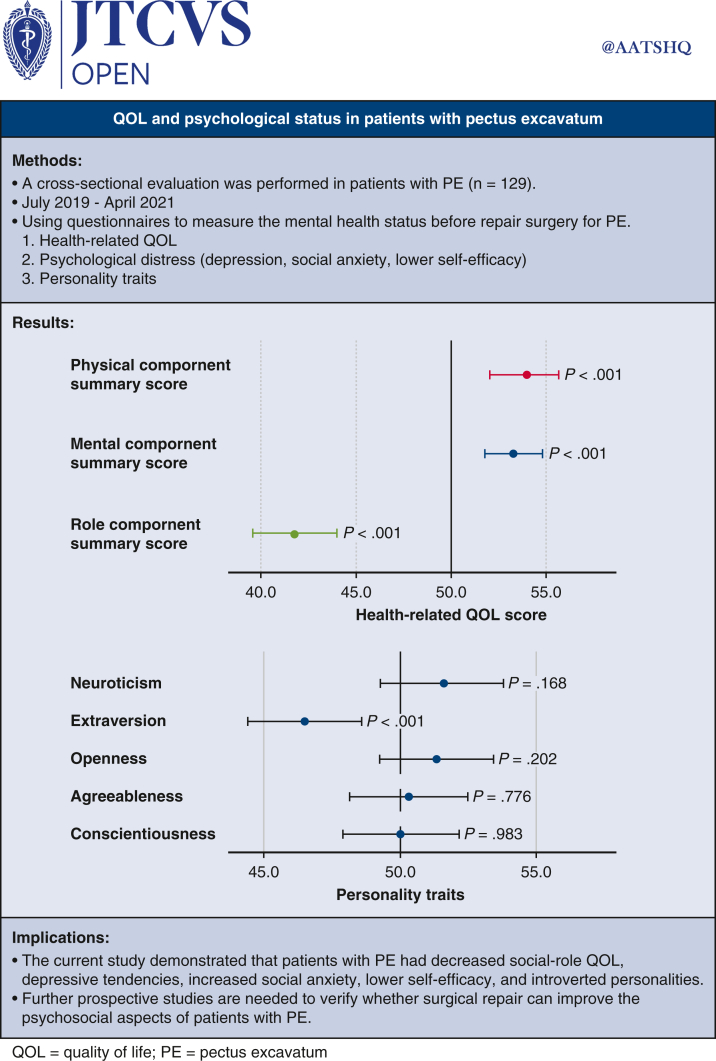
Graphical abstract of the study.

The current study demonstrated that patients with PE had decreased social-role QOL, increased level of social anxiety, lower level of self-efficacy, and introversion. Further prospective studies to evaluate their causal relationship and verify whether surgical repair of PE would lead to improvement of the psychosocial aspects in patients with PE are warranted. See Figure 3 for a graphical abstract of the study.

## Conflict of Interest Statement

The authors reported no conflicts of interest.

The *Journal* policy requires editors and reviewers to disclose conflicts of interest and to decline handling or reviewing manuscripts for which they may have a conflict of interest. The editors and reviewers of this article have no conflicts of interest.

## Figures and Tables

**Table E1:** Comparison of clinical characteristics of included and excluded patients

Variables	Included (n = 129)	Excluded (n = 2)	*P* value
	Mean ± SD, n (%)	Mean ± SD, n (%)	
Age at surgery, y	24.6 ± 11.5	21.0 ± 11.3	.663
Sex			
Male	108 (83.7%)	2 (100.0%)	.533
Female	21 (16.3%)	0 (0%)	
Subjective symptoms	64 (49.6%)	1 (50%)	.991
Smoking	15 (11.6%)	0 (0%)	.607
Family history of pectus excavatum	39 (30.2%)	0 (0%)	.353
ECG abnormalities	63 (48.8%)	0 (0%)	.170
Pulmonary function testing			
VC, mL	3850 ± 30	3470 ± 330	.570
FEV1.0, mL	3240 ± 710	3170 ± 610	.888
Haller index	5.31 ± 2.90	3.31 ± 0.08	.331
Asymmetry depression	63 (48.8%)	0 (0%)	.170
Scoliosis	10 (7.8%)	0 (0%)	.666
Psychiatric disorder	7 (5.4%)	0 (0%)	.735

*SD*, Standard deviation; *ECG*, electrocardiogram; *VC*, vital capacity; *FEV1.0*, forced expiratory volume in 1 second.

**Table E2:** Correlation between Haller index and questionnaire results

Parameters	r value	*P* value
SF-12		
Subscales		
Physical functioning	−0.017	.845
Role physical	0.068	.443
Bodily pain	−0.005	.955
General health	−0.110	.217
Vitality	−0.028	.752
Social functioning	0.077	.384
Role emotional	0.089	.316
Mental health	0.083	.349
Summary scores		
PCS	−0.142	.109
MCS	0.030	.734
RCS	0.106	.230
PHQ-9	0.092	.300
LSAS	0.042	.636
GSES	0.042	.639
NEO-FFI		
Neuroticism	0.016	.853
Extraversion	−0.039	.659
Openness	−0.019	.835
Agreeableness	0.070	.434
Conscientiousness	0.076	.394

*SF-12*, 12-Item Short Form Health Survey; *PCS*, physical component summary score; *MCS*, mental component summary score; *RCS*, role component summary score; *PHQ*, Patient Health Questionnaire; *LSAS*, Liebowitz Social Anxiety Scale; *GSES*, General Self-Efficacy Scale; *NEO-FFI*, NEO Five-Factor Inventory.

**Table E3:** Comparison of questionnaire results with or without symptoms

Parameters	Present (n = 64)	Absence (n = 65)	*P* value
	Mean ± SD	Mean ± SD	
SF-12			
Subscales			
Physical functioning	50.6 ± 11.5	51.9 ± 9.6	.485
Role physical	45.0 ± 11.4	46.2 ± 12.7	.591
Bodily pain	51.7 ± 9.9	52.0 ± 9.5	.881
General health	55.3 ± 10.8	56.2 ± 9.5	.602
Vitality	49.3 ± 9.7	49.9 ± 9.8	.696
Social functioning	49.8 ± 11.1	49.0 ± 11.8	.702
Role emotional	44.4 ± 12.2	45.3 ± 14.0	.689
Mental health	49.5 ± 10.3	48.4 ± 10.5	.523
Summary scores			
PCS	52.9 ± 12.2	55.0 ± 8.3	.276
MCS	53.6 ± 9.0	53.0 ± 8.7	.686
RCS	41.9 ± 12.3	41.7 ± 13.4	.938
PHQ-9	4.56 ± 3.79	4.46 ± 4.15	.886
LSAS	41.0 ± 23.6	44.2 ± 25.9	.463
GSES	44.3 ± 10.9	47.9 ± 11.7	.074
NEO-FFI			
Neuroticism	52.2 ± 13.9	51.0 ± 11.6	.600
Extraversion	44.5 ± 12.6	48.5 ± 10.7	.053
Openness	53.1 ± 12.7	49.6 ± 10.9	.090
Agreeableness	48.7 ± 12.7	51.9 ± 12.0	.136
Conscientiousness	48.3 ± 13.1	51.7 ± 11.0	.116

*SD*, Standard deviation; *SF-12*, 12-Item Short Form Health Survey; *PCS*, physical component summary score; *MCS*, mental component summary score; *RCS*, role component summary score; *PHQ*, Patient Health Questionnaire; *LSAS*, Liebowitz Social Anxiety Scale; *GSES*, General Self-Efficacy Scale; *NEO-FFI*, NEO Five-Factor Inventory.

**Table E4:** Comparison of questionnaire results with or without psychiatric disorder

Parameters	Present (n = 7)	Absence (n = 122)	*P* value
	Mean ± SD	Mean ± SD	
SF-12			
Subscales			
Physical functioning	48.2 ± 15.0	51.4 ± 10.3	.433
Role physical	43.9 ± 15.4	45.7 ± 11.9	.709
Bodily pain	50.9 ± 8.8	51.9 ± 9.7	.796
General health	44.6 ± 10.9	56.4 ± 9.7	.002
Vitality	42.4 ± 4.8	50.0 ± 9.8	.004
Social functioning	40.2 ± 17.3	49.9 ± 10.9	.191
Role emotional	25.9 ± 18.9	46.0 ± 11.9	.031
Mental health	39.8 ± 15.9	49.5 ± 9.8	.015
Summary scores			
PCS	56.2 ± 16.5	53.8 ± 10.1	.563
MCS	45.3 ± 9.4	53.8 ± 8.6	.013
RCS	30.3 ± 20.5	42.4 ± 12.1	.169
PHQ-9	9.14 ± 4.4	4.25 ± 3.8	.001
LSAS	52.4 ± 24.4	42.0 ± 24.7	.281
GSES	36.4 ± 4.5	46.7 ± 11.4	.015
NEO-FFI			
Neuroticism	64.3 ± 6.7	50.8 ± 12.7	.064
Extraversion	35.7 ± 4.4	47.1 ± 11.8	.014
Openness	52.1 ± 10.2	51.3 ± 12.1	.440
Agreeableness	47.0 ± 11.3	50.5 ± 12.5	.864
Conscientiousness	50.7 ± 6.0	50.0 ± 12.4	.075

*SD*, Standard deviation; *SF-12*, 12-Item Short Form Health Survey; *PCS*, physical component summary score; *MCS*, mental component summary score; *RCS*, role component summary score; *PHQ*, Patient Health Questionnaire; *LSAS*, Liebowitz Social Anxiety Scale; *GSES*, General Self-Efficacy Scale; *NEO-FFI*, NEO Five-Factor Inventory.

**Table E5:** Correlation between pulmonary function (VC and FEV1.0) and questionnaire results

Parameters	VC		FEV1.0	
	r value	*P* value	r value	*P* value
SF-12				
Subscales				
Physical functioning	0.079	.397	0.111	.234
Role physical	0.134	.152	0.124	.184
Bodily pain	0.133	.155	0.121	.194
General health	0.094	.318	0.053	.571
Vitality	0.041	.659	0.024	.798
Social functioning	0.099	.290	0.063	.502
Role emotional	−0.012	.898	−0.056	.553
Mental health	0.024	.797	0.001	.988
Summary scores				
PCS	0.126	.177	0.154	.098
MCS	0.033	.724	−0.001	.989
RCS	0.024	.795	−0.009	.922
PHQ-9	−0.141	.131	−0.084	.370
LSAS	−0.144	.122	−0.117	.211
GSES	−0.019	.842	−0.064	.497
NEO-FFI				
Neuroticism	0.032	.731	0.058	.539
Extraversion	−0.050	.597	−0.093	.322
Openness	0.272	.003	0.210	.024
Agreeableness	−0.122	.193	−0.103	.273
Conscientiousness	−0.011	.909	−0.038	.683

*VC*, Vital capacity; *FEV1.0*, forced expiratory volume in 1 second; *SF-12*, 12-Item Short Form Health Survey; *PCS*, physical component summary score; *MCS*, mental component summary score; *RCS*, role component summary score; *PHQ*, Patient Health Questionnaire; *LSAS*, Liebowitz Social Anxiety Scale; *GSES*, General Self-Efficacy Scale; *NEO-FFI*, NEO Five-Factor Inventory.

**Table E6:** Comparison of questionnaire results with or without electrocardiogram abnormality

Parameters	Present (n = 63)	Absence (n = 66)	*P* value
	Mean ± SD	Mean ± SD	
SF-12			
Subscales			
Physical functioning	51.5 ± 10.9	50.9 ± 10.3	.743
Role physical	45.5 ± 11.8	45.6 ± 12.4	.971
Bodily pain	52.4 ± 7.9	51.4 ± 11.1	.576
General health	56.7 ± 9.8	54.9 ± 10.5	.334
Vitality	49.2 ± 9.8	50.0 ± 9.6	.604
Social functioning	50.0 ± 11.2	48.8 ± 11.7	.533
Role emotional	46.9 ± 13.5	42.9 ± 12.5	.084
Mental health	49.1 ± 10.1	48.8 ± 10.7	.855
Summary scores			
PCS	54.2 ± 9.8	53.8 ± 11.1	.829
MCS	53.2 ± 8.4	53.4 ± 9.3	.910
RCS	42.9 ± 13.3	40.7 ± 12.3	.340
PHQ-9	4.51 ± 4.07	4.52 ± 3.88	.992
LSAS	42.1 ± 25.9	43.0 ± 23.8	.837
GSES	46.5 ± 12.1	45.7 ± 10.7	.705
NEO-FFI			
Neuroticism	49.9 ± 13.4	53.2 ± 12.1	.148
Extraversion	45.6 ± 11.8	47.4 ± 11.8	.403
Openness	53.3 ± 13.0	49.4 ± 10.6	.064
Agreeableness	48.9 ± 10.7	51.7 ± 13.8	.201
Conscientiousness	48.4 ± 11.4	51.6 ± 12.7	.127

*SD*, Standard deviation; *SF-12*, 12-Item Short Form Health Survey; *PCS*, physical component summary score; *MCS*, mental component summary score; *RCS*, role component summary score; *PHQ*, Patient Health Questionnaire; *LSAS*, Liebowitz Social Anxiety Scale; *GSES*, General Self-Efficacy Scale; *NEO-FFI*, NEO Five-Factor Inventory.

**Table E7:** Comparison of questionnaire results with or without asymmetry depression

Parameters	Present (n = 63)	Absence (n = 66)	*P* value
	Mean ± SD	Mean ± SD	
SF-12			
Subscales			
Physical functioning	52.6 ± 9.4	49.9 ± 11.4	.150
Role physical	48.0 ± 9.9	43.3 ± 13.5	.026
Bodily pain	52.0 ± 10.2	51.7 ± 9.2	.877
General health	57.2 ± 8.5	54.4 ± 11.4	.120
Vitality	49.7 ± 9.9	49.5 ± 9.6	.891
Social functioning	50.8 ± 10.3	48.1 ± 12.4	.184
Role emotional	46.9 ± 10.3	42.9 ± 15.1	.082
Mental health	50.5 ± 9.4	47.5 ± 11.1	.106
Summary scores			
PCS	54.8 ± 9.1	53.2 ± 11.7	.392
MCS	53.4 ± 8.1	53.2 ± 9.5	.930
RCS	44.0 ± 11.0	39.6 ± 14.1	.052
PHQ-9	3.52 ± 3.49	5.45 ± 4.17	.005
LSAS	38.3 ± 23.7	46.7 ± 25.2	.053
GSES	47.3 ± 11.3	44.9 ± 11.4	.232
NEO-FFI			
Neuroticism	51.8 ± 12.9	51.4 ± 12.8	.860
Extraversion	46.2 ± 12.1	46.8 ± 11.6	.764
Openness	52.7 ± 12.5	50.0 ± 11.3	.203
Agreeableness	51.3 ± 12.4	49.4 ± 12.4	.384
Conscientiousness	49.1 ± 13.2	50.9 ± 11.1	.424

*SD*, Standard deviation; *SF-12*, 12-Item Short Form Health Survey; *PCS*, physical component summary score; *MCS*, mental component summary score; *RCS*, role component summary score; *PHQ*, Patient Health Questionnaire; *LSAS*, Liebowitz Social Anxiety Scale; *GSES*, General Self-Efficacy Scale; *NEO-FFI*, NEO Five-Factor Inventory.

**Table E8:** Comparison of questionnaire results with or without scoliosis

Parameters	Present (n = 11)	Absence (n = 118)	*P* value
	Mean ± SD	Mean ± SD	
SF-12			
Subscales			
Physical functioning	54.5 ± 4.0	50.9 ± 10.9	.029
Role physical	48.2 ± 11.4	45.3 ± 12.2	.448
Bodily pain	53.3 ± 7.5	51.7 ± 9.8	.620
General health	55.7 ± 11.2	55.8 ± 10.1	.971
Vitality	50.0 ± 10.0	49.6 ± 9.7	.877
Social functioning	53.5 ± 7.4	49.0 ± 11.7	.220
Role emotional	45.2 ± 15.8	44.8 ± 12.9	.927
Mental health	51.8 ± 7.1	48.7 ± 10.6	.342
Summary scores			
PCS	55.0 ± 4.9	53.9 ± 10.8	.732
MCS	53.8 ± 6.4	53.3 ± 9.0	.859
RCS	44.6 ± 11.7	41.5 ± 12.9	.446
PHQ-9	4.00 ± 4.69	4.56 ± 3.90	.656
LSAS	41.6 ± 28.5	42.7 ± 24.5	.894
GSES	49.0 ± 10.5	45.8 ± 11.5	.380
NEO-FFI			
Neuroticism	51.4 ± 10.2	51.6 ± 13.0	.958
Extraversion	44.3 ± 8.2	46.7 ± 12.1	.516
Openness	51.8 ± 13.7	51.3 ± 11.8	.890
Agreeableness	48.9 ± 13.0	50.4 ± 12.4	.696
Conscientiousness	57.0 ± 8.9	49.4 ± 12.2	.046

*SD*, Standard deviation; *SF-12*, 12-Item Short Form Health Survey; *PCS*, physical component summary score; *MCS*, mental component summary score; *RCS*, role component summary score; *PHQ*, Patient Health Questionnaire; *LSAS*, Liebowitz Social Anxiety Scale; *GSES*, General Self-Efficacy Scale; *NEO-FFI*, NEO Five-Factor Inventory.
